# Parental perceptions and experiences of infant crying: A systematic review and synthesis of qualitative research

**DOI:** 10.1111/jan.15492

**Published:** 2022-11-14

**Authors:** Ingrid Muller, Daniela Ghio, Jasmine Mobey, Hannah Jones, Samantha Hornsey, Amy Dobson, Emma Maund, Miriam Santer

**Affiliations:** ^1^ Primary Care Research Centre University of Southampton Southampton UK; ^2^ Department of Psychology University of Manchester Manchester UK

**Keywords:** excessive crying, infant crying, literature review, qualitative, systematic review, thematic synthesis

## Abstract

**Background:**

Excessive infant crying is common and can have a huge impact on families and well‐being. Systematically reviewing qualitative studies on infant crying can provide a greater understanding of parental perceptions and experiences.

**Aim:**

This study sought to systematically review and thematically synthesize qualitative studies exploring parents/carers' views and experiences of infant crying.

**Design:**

A systematic review and synthesis of qualitative research.

**Data Sources:**

Electronic databases MEDLINE, EMBASE, PsycINFO and CINAHL were searched from the earliest date available to January 2022. We selected papers focussing on parents/carers' experiences, views, attitudes and beliefs about infant crying. We excluded papers focussing on health professionals' views and children older than 12 months.

**Review Methods:**

Thematic synthesis was followed for the analysis of included studies and quality appraisal was conducted.

**Results:**

We synthesized 22 papers, reporting data from 376 participants in eight countries. Four analytical themes were developed: (1) Experiences and impact of crying; (2) parental management strategies; (3) the role of the health professional; (4) the role of infant feeding and maternal diet. Our findings suggest that infant crying has a substantial emotional impact on parents/carers that often impacts relationships. Parents/carers reported using a range of soothing techniques and coping strategies but were desperate to find effective treatment or cure. Support was often perceived as lacking. Excessive crying and beliefs about the role of maternal diet on breastmilk were reported to undermine parents' confidence in breastfeeding by making them feel their milk is insufficient or harmful, or through pressure from others to stop breastfeeding.

**Conclusion:**

Parents/carers use a range of strategies to interpret and deal with the challenges of infant crying, but there is a need for more information and support.

**Impact:**

Findings can be used to inform future research and interventions to support families experiencing excessive infant crying.

## 1 INTRODUCTION

Excessive crying affects around 20% of infants in the first months of life (Wake et al., 2006; Wolke et al., 2017) and can have a substantial impact on families including parental anxiety and depression, early cessation of breastfeeding, increased risk of non‐accidental injury and child behavioural difficulty (Douglas & Hill, 2011). Normal infant crying is high across the first 6 weeks of life and can reach a peak of over 2 h a day before reducing drastically between 6 and 12 weeks of age to 1 h a day (Wolke et al., 2017). There is clear evidence that caring for an excessively crying infant is harmful to relationships and health, but more research is needed to understand this complex phenomenon. Synthesizing findings from existing qualitative research can provide valuable new insights into parents' views, experiences and management of infant crying and how these may relate to feeding decisions.

## 2 BACKGROUND

Excessive infant crying of unknown cause, also known as infantile colic, is defined as uncontrollable crying for more than 3 h a day, for more than 3 days a week, for at least 1 week, in a healthy infant up to 4 months of age (NHS, 2022). Colic is estimated to affect 17%–25% of infants in the first 6 weeks of life (Wolke et al., 2017). Despite its high prevalence, the aetiology of colic is very poorly understood, and diagnosis usually requires ruling out other possible causes such as gastrointestinal or feeding problems, or infections (Douglas & Hill, 2011). The severity of infant crying itself is thought to occur along a continuum, which depending on the definition of excessive crying can affect up to 12% of infants (Reijneveld et al., 2001). There is evidence of an association between excessive infant crying and poorer long‐term outcomes including impact on the parent–child relationship, child mood disorders and possible developmental concerns (DeGangi et al., 2000; Hemmi et al., 2011). These may be mediated by parental attributions and perceptions of their crying infant (James‐Roberts, 2001; Smarius et al., 2017), and early intervention to support parents affected by excessive infant crying could help mitigate these potential negative outcomes (Gilkerson et al., 2020).

Infant crying has been found to have a major impact on parents and families. It is associated with parental anxiety, maternal and paternal depression (McMahon et al., 2001; Smart & Hiscock, 2007), high levels of health service use and is the biggest risk factor for infant abuse, particularly shaken baby syndrome (Reijneveld et al., 2004). A mixed‐methods literature review focussing on the impact of excessive infant crying on the family found consequences included: feelings of desperation, impaired breastfeeding, ‘ruined’ everyday life, isolation and loneliness, strained and broken family relationships, physical and mental exhaustion and feelings of failure as a parent (Botha et al., 2019). The current review aims to build on these findings by exploring the qualitative literature beyond the impact of excessive crying to further understand parental behaviours.

## 3 THE REVIEW

### 3.1 Aim

We aimed to conduct a systematic review and thematic synthesis of the qualitative literature on parents/carers' views and experiences of infant crying. For the remainder of this paper parents and carers are collectively referred to as parents.

### 3.2 Design

We conducted a systematic review and thematic synthesis (Thomas & Harden, 2008) of qualitative papers. Enhancing transparency in reporting the synthesis of qualitative research (ENTREQ) statement (Tong et al., 2012) was used to facilitate reporting.

### 3.3 Search methods

Four electronic databases were first searched on 6th October 2020 using a comprehensive search strategy (Table S1) devised using the SPIDER search strategy tool (Cooke et al., 2012): Medline (1946–2020), EMBASE (1947–2020), PsychINFO (1806–2020), CINAHL (1981–2020). Reference lists of all included papers were checked to minimize the risk of missing key papers. Two authors (J.M. and H.J.) independently screened all titles and abstracts against the inclusion criteria and any discrepancies were discussed with M.S. and I.M. The searches were updated on 28 January 2022, and all new titles and abstracts were screened independently by I.M. and S.H.

Papers were included where the primary focus was parents' views and experiences of infant crying. A recent analysis of online parenting forum discussions found that diagnostic labels are used interchangeably and not well understood (Ghio et al., 2022). All parental experiences of infant crying were therefore included in the review, and we did not limit inclusion to infants with a diagnostic label of colic or excessive crying. To be eligible, papers must have reported the use of qualitative methods for data collection and analysis, and at least half of the reported results had to relate to infant crying. Papers were excluded where the focus was not on a generalized parent population, or where the study focussed on a single treatment modality for crying. There were no date or language restrictions. See Table 1 for inclusion and exclusion criteria.

**TABLE 1:** Eligibility criteria for included articles

	Inclusion criteria	Exclusion criteria
Sample	Parents and carers of infants	Health professionals' views only
		Not generalized parent population (e.g. only parents with an anxiety disorder)
		Focus on children older than 12 months
Phenomenon of interest	Infant crying	Single treatment modality for crying (e.g. acupuncture)
Design	Qualitative (including ethnography, grounded theory, phenomenology, focus groups, interviews and participant observations)	Case studies Quantitative studies Literature reviews Experimental design
Evaluation	Reports of parental experiences, views, attitudes, beliefs	
Research type	Qualitative, Mixed methods	Studies that do not report qualitative data collection and qualitative data

### 3.4 Search outcome

**FIGURE 1:**
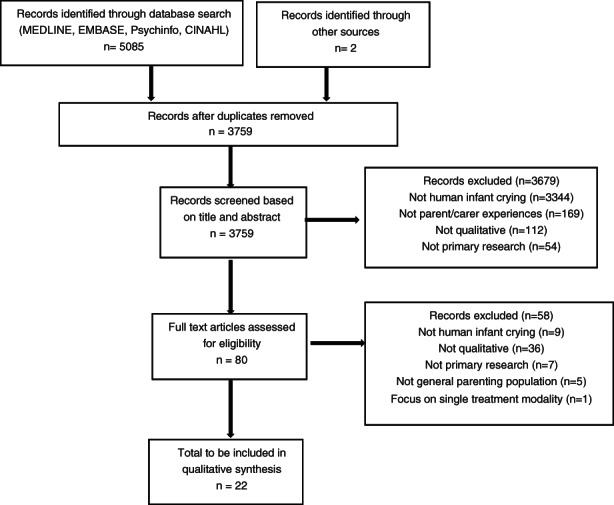
CONSORT diagram

Database searches yielded 5085 results and two were identified through reviewing reference lists. Deduplication resulted in 3759, of which 80 were assessed as potentially eligible following screening titles and abstracts. Full‐text papers were obtained for these 80 to further assess eligibility, resulting in 22 papers being included in the final systematic review and synthesis (see Figure 1).

### 3.5 Quality appraisal and data extraction

Key study characteristics were extracted from each paper. These included: first author, publication year, focus, number of participants, country, setting, data collection methodology, data analysis methodology and key themes reported by authors. Reporting quality of included papers was appraised by J.M. or S.H. using the CASP checklist (Singh, 2013) for qualitative studies. Quality appraisal and data extraction were discussed with IM and MS. No studies were excluded as a result of the quality appraisal. Reporting standards have changed since the 1980s and including older papers to explore how parental perceptions have changed over time was deemed valuable in this current review. All included studies were high quality except for Thompson 1986 (Thompson et al., 1986), which was rated as poor quality (Table S2).

### 3.6 Data synthesis

Thematic Synthesis (Braun & Clarke, 2019) was followed for the analysis of included studies. First, data were explored and described. The texts were read and re‐read before the data were inductively coded according to content and meaning to produce an initial coding framework of descriptive themes. Key findings and quotes from each paper were listed and tabulated so that they could be explored and compared. The analysis also drew on principles from meta‐ethnography (Noblit et al., 1988) by using the notion of first‐, second‐ and third‐order constructs to synthesize qualitative papers. First‐order constructs refer to direct participant data, second‐order constructs are the original researchers' interpretations of the data, and third‐order constructs are the new interpretations arising from the synthesis of second‐order constructs from multiple papers. The initial coding frame was developed by J.M. and M.S. and iteratively refined through discussions with I.M., M.S. and D.G. to offer different interpretations of the data and facilitate the generation of analytic themes that go beyond describing findings from included papers.

## 4 RESULTS

### 4.1 Study characteristics

Included studies were conducted between 1986 and 2020 in USA (*n* = 11), UK (*n* = 3), Switzerland (*n* = 2), Sweden (*n* = 2), South Africa(*n* = 1), Canada (*n* = 1), Norway (*n* = 1) and Vietnam (*n* = 1) and reported data from qualitive interviews with 376 parents. Participants were mostly mothers but also included fathers and grandmothers. Studies included infants who had been given diagnostic labels of colic (*n* = 10), excessive, persistent or inconsolable crying (*n* = 5) or no diagnostic label (*n* = 7). See Table 2 for characteristics of included studies.

**TABLE 2:** Characteristics of included studies

No.	Authors (year)	Country	Participants (n)	Infant diagnostic label	Principle views/experiences explored	Method of data collection	Method of data analysis
1	Thompson et al. (1986)	USA	Parents and HCPs (50)	Colic—HCP diagnosed	The processes associated with infant colic in the family	Interview	Not given
2	Wiley et al. (2020)	USA	Parents (25)	None, parents of newborns	Parental perceptions of infant crying and abusive head trauma	Semi‐structured interviews	Thematic analysis
3	Wade et al. (2005)	USA	Mothers (7)	None, mothers at risk of AHT	Impact of crying on mothers at high risk of abusing their child	Focus group	Phenomenological Approach
4	Landgren et al. (2012)	Sweden	Mothers (10) and fathers (7)	Colic—HCP diagnosed	Parents' experience of infant colic	Semi‐structured interviews and a focus group	Content analysis
5	Cox and Roos (2008)	South Africa	Not given	Colic—HCP diagnosed	Experiences of first‐time mothers with colicky infants	Semi‐structured interviews	Thematic analysis
6	Drummond et al. (1993)	Canada	Mothers (19)	None, mothers of newborns	Views and understanding of infant crying and soothing	Semi‐structured interviews	Thematic analysis
7	Kurth et al. (2010)	Switzerland	Mothers (15)	None, mothers of newborns	Mother's experiences and responses to infant crying	Semi‐structured interviews, observation	Phenomenological Approach
8	Landgren and Hallström (2011)	Sweden	Mothers (12) and fathers (11)	Colic—HCP diagnosed	The meaning of being a parent of a colicky infant	Semi‐structured interviews	Phenomenological approach
9	Megel et al. (2011)	USA	Mothers (13)	Persistent crying—parent report	Experiences of parenting an irritable infant	Semi‐structured interviews	Grounded theory
10	Murray et al. (2018)	Vietnam	Mothers (21), grandmothers (3)	None, mothers of newborns	Caregivers' understanding of, and responses to, unsettled infant behaviour	Semi‐structured interviews	Thematic analysis
11	Poskey and Hersch (2012)	USA	Mothers (3), fathers (3)	Inconsolable infant crying ‐ parent report	Parental experience of infant crying	Semi‐structured interviews	Thematic analysis
12	Poskey et al. (2014)	USA	Mothers (2), fathers (2)	Inconsolable infant crying—parent report	Parents' thoughts, feelings, behaviour and actions towards an inconsolable infant	Questionnaire, home observation	Ethnography
13	Ellett et al. (2009)	USA	Fathers (10)	Colic ‐ parent report	Fathers' experiences living with colicky infant	Semi‐structured interviews	Interpretive phenomenological analysis
14	Keefe and Froese‐Fuetz (1991)	USA	Mothers (15)	Colic ‐ HCP diagnosed	Experiences of mothers caring for an irritable infant	Unstructured interviews	Thematic analysis
15	Kidd et al. (2019)	Norway	Mothers (21)	Colic ‐ parent report	Mothers' perceptions of the role of maternal diet on crying in infant	Semi‐structured interviews and focus groups	Content analysis
16	Ellett et al. (2005)	USA	Parents (44)	Colic ‐ parent report	Experiences living with colicky infant	Semi‐structured interviews	Thematic analysis
17	Nash et al. (2008)	UK	Mothers (22), fathers (2)	None, parents attending child health clinic	Views of the crying behaviour of infants	Semi‐structured interviews	Thematic analysis
18	Kurth et al. (2014)	Switzerland	Mothers (15)	None, mothers of newborns	Views and experiences of first‐time and experienced mothers of infant crying	Semi‐structured interviews and observation	Phenomenological Approach
19	Long and Johnson (2001)	UK	Parents (25)	Excessive crying – parent report	Parental experience of coping with excessively crying infant	Semi‐structured interviews and observation	Grounded theory
20	Ellett et al. (2005)	USA	Parents (15)	Colic ‐ parent report	Perceptions of lasting effects of colic	Semi‐structured interviews	Thematic analysis
21	Oaten et al. (2019)	UK	Mothers (6)	Excessive crying—parent report	Parental experience of coping with excessively crying infant	Semi‐structured interviews	Thematic analysis
22	Levitzky and Cooper (2000)	USA	Mothers (23)	Colic—HCP diagnosed	The impact of infant colic on the emotional state of the mother	Structured interviews	Not stated

Most interviews were conducted face‐to‐face, but some were by telephone and one study included email interviews. The most commonly reported method of data analysis was thematic analysis. Most studies focussed on parental experiences and the impact of infant crying, some focussed on parental understanding and responses to infant crying, and one paper focussed on parental beliefs about the role of diet on infant crying.

### 4.2 Thematic synthesis

**FIGURE 2:**
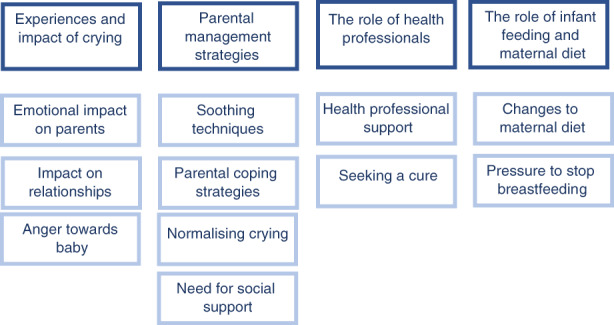
Overview of themes

Line‐by‐line coding generated descriptive themes, shown in Figure 2, which were then further developed into four overarching analytical themes: (1) Experiences and impact of crying. (2) Parental management strategies. (3) The role of health professionals. (4) The role of feeding/diet. The number of themes emerging from individual papers included in this review ranged from 2 to 9. None of the included papers covered all the themes identified in this review. Where findings emerged from largely one population, this is highlighted in the relevant section. See Table 3 for information on how the themes map to each paper.

**TABLE 3:** Themes identified in each study

Themes	Study reference																					
	1	2	3	4	5	6	7	8	9	10	11	12	13	14	15	16	17	18	19	20	21	22
Experiences and impact of crying																						
Emotional impact on parents	✓	✓	✓	✓	✓	—	✓	✓	✓	✓	✓	✓	✓	✓	—	✓	✓	✓	✓	✓	✓	✓
Impact on relationships	—	—	—	✓	✓	—	—	✓	—	✓	—	—	✓	—	—	—	—	✓	✓	✓	✓	✓
Anger towards baby	✓	—	—	✓	✓	—	✓	—	✓	—	—	✓	—	—	—	✓	✓	—	—	—	✓	✓
Parental management strategies																						
Soothing techniques	✓	✓	✓	—	✓	✓	✓	✓	✓	✓	✓	✓	—	✓	—	—	—	✓	—	—	—	—
Parental coping strategies	✓	✓	✓	—	—	✓	✓	✓	✓	✓	✓	✓	—	✓	—	✓	✓	✓	✓	—	✓	—
Need for social support	—	✓	✓	✓	✓	—	—	✓	✓	✓	—	—	✓	✓	✓	—	✓	—	✓	✓	✓	—
Normalizing crying	✓	✓	✓	✓	✓	—	✓	✓	—	✓	—	✓	✓	✓	✓	✓	✓	—	—	✓	✓	—
The role of health professionals																						
Health professional support	✓	—	✓	✓	✓	—	✓	✓	✓	—	—	—	✓	—	✓	—	—	—	✓	✓	✓	✓
Seeking a cure	✓	—	—	✓	✓	✓	—	✓	✓	✓	✓	✓	—	✓	✓	—	—	—	✓	—	✓	—
The role of infant feeding and maternal diet																						
Changes to maternal diet	✓	—	—	—	—	—	—	✓	✓	—	—	—	—	—	✓	—	—	—	—	—	—	✓
Pressure to stop breastfeeding	—	—	—	—	—	—	—	—	✓	✓	—	—	—	✓	✓	—	—	—	—	—	—	—

#### 4.2.1 Experiences and impact of crying

All papers discussed parental experiences of infant crying and the impact it had on their lives. The impact of crying was described in terms of emotional impact, impact on relationships, and feeling anger towards the baby.

##### Emotional impact of crying

The vast majority of included studies reported accounts of parents experiencing significant emotional impact from infant crying (Cox & Roos, 2008; Ellett et al., 2009, 2005; Ellett & Swenson, 2005; Keefe & Froese‐Fuetz, 1991; Kurth et al., 2014, 2010; Landgren & Hallstrom, 2011; Landgren et al., 2012; Levitzky & Cooper, 2000; Long & Johnson, 2001; Megel et al., 2011; Murray et al., 2018; Nash et al., 2008; Oaten & Miller, 2019; Poskey & Hersch, 2012; Poskey et al., 2014; Thompson et al., 1986; Wade et al., 2005; Wiley et al., 2020). The two included studies where this subtheme did not emerge focussed specifically on soothing techniques (Drummond et al., 1993) and dietary restrictions resulting from infant colic (Kidd et al., 2019). Parents described feeling worried, anxious, and distressed, and one study described a mother's experience of needing counselling to cope with the distress caused by her baby's colic (Ellett & Swenson, 2005). In most studies, parents also discussed exhaustion from lack of sleep (Cox & Roos, 2008; Drummond et al., 1993; Ellett & Swenson, 2005; Ellett et al., 2005, 2009; Kurth et al., 2010; Landgren & Hallstrom, 2011; Levitzky & Cooper, 2000; Long & Johnson, 2001; Megel et al., 2011; Murray et al., 2018; Nash et al., 2008; Thompson et al., 1986).

Both anger and frustration and also, partly, sadness came. So, yes, a combination of helplessness, sadness and pure rage and … yes, madness. I am constantly furious (Landgren et al., 2012)

Tiredness. In your head. So much tiredness… When my husband came home yesterday, I sat with her in my arms, just crying. I had been crying since she stopped crying… I'm totally drained. (Landgren & Hallstrom, 2011)

All studies that described the emotional impact of crying reported parents experiencing feelings of frustration, failure, inadequacy and self‐blame for being unable to soothe their baby and manage their crying (Cox & Roos, 2008; Ellett & Swenson, 2005; Ellett et al., 2005, 2009; Keefe & Froese‐Fuetz, 1991; Kurth et al., 2014, 2010; Landgren & Hallstrom, 2011; Landgren et al., 2012; Levitzky & Cooper, 2000; Long & Johnson, 2001; Megel et al., 2011; Murray et al., 2018; Nash et al., 2008; Oaten & Miller, 2019; Poskey & Hersch, 2012; Poskey et al., 2014; Thompson et al., 1986; Wade et al., 2005; Wiley et al., 2020).

Upset and angry with myself you know it's my fault I don't know it's just natural isn't it like any mother really don't know what to do. I just want to make him better — you know, stop him crying. (Nash et al., 2008)

It truly changed who I am. Life was guilt, anger, frustration, confusion, sadness, and the feeling of having been cheated. (Ellett et al., 2005)

In some studies, parents described how feelings of failure over time became feelings of hopelessness that the crying would ever improve, and helplessness that there was nothing they could do to improve it (Cox & Roos, 2008; Landgren et al., 2012; Oaten & Miller, 2019)

I felt that it would never end, the total utter helplessness to the point where you feel that you can't make this better and then losing hope that it would ever come to an end. (Cox & Roos, 2008)

##### Impact on relationships

A recurring theme across many studies was parents' descriptions of the adverse effect having a baby with excessive crying had on their relationships with their partners (Cox & Roos, 2008; Ellett et al., 2005, 2009; Kurth et al., 2014; Landgren & Hallstrom, 2011; Landgren et al., 2012; Levitzky & Cooper, 2000; Long & Johnson, 2001; Murray et al., 2018; Oaten & Miller, 2019).

It's a loveless marriage with a relationship that won't exist until the crying stops, if even then, and we argue about it all the time. (Long & Johnson, 2001)

He is so angry. Every day it gets worse. It's killing us. If we had an easy baby, there would be no problem. (Levitzky & Cooper, 2000)

This was often discussed by mothers in relation to feeling alone and unsupported by their partners. A mother in one study (Cox & Roos, 2008) described how her husband's life remained largely unchanged as he was still able to socialize and go out as before whilst her life had been dominated by looking after a baby with colic.

Several papers discussed parents feeling like their parenting was being judged by others, including friends, family, and health professionals (Cox & Roos, 2008; Landgren & Hallstrom, 2011; Landgren et al., 2012; Levitzky & Cooper, 2000; Long & Johnson, 2001; Megel et al., 2011; Oaten & Miller, 2019). This sometimes led to parents isolating themselves from unsolicited advice, negative comments, and perceived judgement. This often emerged as second‐order constructs (Landgren et al., 2012; Levitzky & Cooper, 2000; Long & Johnson, 2001; Oaten & Miller, 2019), for example “Although there were examples of parents being explicitly accused of poor parenting practice, more often the feelings of guilt resulted from self‐recrimination or perhaps from perceptions of external criticism” (Long & Johnson, 2001). However, feeling judged was also supported by first‐order data excerpts from participants.

Then it's the comments from people, from the paediatrician to my friends. There were times when we went to visit them and looked exhausted, and they would say that you could take an antidepressant if YOU'RE not coping. You feel so frustrated because you're trying to explain that anyone in your situation wouldn't be coping (Cox & Roos, 2008).

The worst part is that everyone has something to try. And of course, it's stuff you have already tried, and you know it's not going to work. Finally, you just get tired of explaining it. (Megel et al., 2011)

In some studies, parents also discussed the negative impact infant crying had on the relationship with their child (Cox & Roos, 2008; Ellett & Swenson, 2005; Landgren & Hallström, 2011; Landgren et al., 2012; Levitzky & Cooper, 2000; Megel et al., 2011; Oaten & Miller, 2019). This was usually a result of feeling like a failure and a bad parent or feeling rejected by the child.

I felt guilty and like a bad mother when I could not calm him down. I imagined that he did not love me and one day I told my husband that I was going to stop breastfeeding because the baby was just ‘using me’ and I wasn't going to let him do that. I immediately felt guilty and childish and, of course, did not stop breastfeeding. (Ellett & Swenson, 2005)

What are we doing wrong? My baby is telling me I'm not a good mother. (Megel et al., 2011)

##### Anger towards baby

Participants in nearly half of the included studies expressed feelings of anger and frustration towards their baby as a result of excessive crying (Cox & Roos, 2008; Ellett & Swenson, 2005; Kurth et al., 2010; Landgren et al., 2012; Levitzky & Cooper, 2000; Megel et al., 2011; Nash et al., 2008; Oaten & Miller, 2019; Poskey et al., 2014; Thompson et al., 1986).

There were moments when, both me and my husband … when she was apoplectic and howling so much that I almost got this thought, ‘now I'll take a pillow and put over her face just until she quietens down, until the screaming stops. (Landgren et al., 2012)

Some of the studies where parents expressed feelings of anger towards their child (Levitzky & Cooper, 2000; Nash et al., 2008; Poskey et al., 2014) specifically aimed to explore experiences and triggers to prevent abusive head trauma because of shaking the baby, which can have devastating outcomes. Some papers discussed how aggression can be induced by crying, maternal thoughts of harm and implication in the prevention of head trauma (Landgren et al., 2012; Thompson et al., 1986; Wade et al., 2005; Wiley et al., 2020). The fantasies or thoughts of harm reported in these papers were all from parents of infants who have been labelled as having colic or inconsolable crying.

There were times when you want to throw him against the wall. (Cox & Roos, 2008)

In one paper, most parents said that they could not understand why someone would shake an infant (Wiley et al., 2020). However, this paper studied parents of infants that cried normally (convenience sample of parents of newborns), which could explain these differences.

She can cry for hours. I wouldn't shake her. I wouldn't be able to see myself shaking her. (Wiley et al., 2020)

#### 4.2.2 Parental management strategies

##### Soothing techniques

A range of consistently similar ways to manage crying was reported across the different papers. A common response was to pick the baby up when they were crying. Some papers also reported participants doing basic checks to interpret the reason for the crying or systematically working through potential soothing techniques to stop the crying (Cox & Roos, 2008; Drummond et al., 1993; Keefe & Froese‐Fuetz, 1991; Kurth et al., 2014, 2010; Landgren & Hallström, 2011; Megel et al., 2011; Murray et al., 2018; Poskey & Hersch, 2012; Poskey et al., 2014; Thompson et al., 1986; Wade et al., 2005; Wiley et al., 2020).

I check to see if his diaper is wet, check to see if he is hungry, rub his back. Sometimes I give him a nice warm bath, I walk around with him … I sing to him. (Poskey et al., 2014)

Other approaches to soothe a crying baby were feeding, auditory interventions such as singing or white noise and the use of movement (Drummond et al., 1993; Ellett et al., 2009; Keefe & Froese‐Fuetz, 1991; Kidd et al., 2019; Kurth et al., 2014, 2010; Landgren & Hallström, 2011; Megel et al., 2011; Murray et al., 2018; Poskey & Hersch, 2012; Poskey et al., 2014; Wade et al., 2005).

Potentially harmful strategies, such as medicating the baby with homeopathic pills or antihistamines, were reported by parents in one of the papers. (Ellett & Swenson, 2005).

##### Strategies to help parents cope

Participants in most studies detailed a range of coping strategies they used to help them deal with the demands of their baby's crying (Drummond et al., 1993; Ellett & Swenson, 2005; Keefe & Froese‐Fuetz, 1991; Kurth et al., 2010, 2014; Landgren & Hallström, 2011; Long & Johnson, 2001; Megel et al., 2011; Murray et al., 2018; Nash et al., 2008; Oaten & Miller, 2019; Poskey & Hersch, 2012; Poskey et al., 2014; Thompson et al., 1986; Wade et al., 2005; Wiley et al., 2020). Perhaps the most important was finding ways to block out the sound of crying, taking a break and safety planning. Parents in many studies described putting their baby down and physically walking away when they felt they were becoming too frustrated (Ellett et al., 2009; Kurth et al., 2010; Megel et al., 2011; Nash et al., 2008; Poskey & Hersch, 2012; Wiley et al., 2020).

For sanity's sake I'd just had to put him in his crib and walk away and kind of give myself a little time out to kind of get my breath. (Poskey & Hersch, 2012)

Other coping strategies included measures to directly calm themselves, such as driving (Poskey & Hersch, 2012), singing, smoking, taking anti‐anxiety medication (Wade et al., 2005) and even boxing (Poskey & Hersch, 2012). Cognitive strategies included “keeping a stiff upper lip” and persevering (Wade et al., 2005).

Sometimes there is no one around; I just have to buck up and deal with it. (Wade et al., 2005)

One paper described how parents used self‐talk, telling themselves over and over “it's only colic and it can't last forever” (Megel et al., 2011). Some parents also adapted their expectations of what being a parent would be like (Drummond et al., 1993; Kurth et al., 2010, 2014; Thompson et al., 1986). Considering the positives in the situation, such as feeling gratitude the baby was healthy, was another coping method in some papers (Drummond et al., 1993; Ellett & Swenson, 2005; Kurth et al., 2014; Thompson et al., 1986; Wade et al., 2005).

##### Need for social support

Participants in the majority of studies discussed the importance of social support for managing infant crying (Cox & Roos, 2008; Ellett et al., 2009; Keefe & Froese‐Fuetz, 1991; Kidd et al., 2019; Landgren & Hallström, 2011; Landgren et al., 2012; Long & Johnson, 2001; Megel et al., 2011; Murray et al., 2018; Nash et al., 2008; Oaten et al., 2019; Wade et al., 2005; Wiley et al., 2020).

You gotta have a support system, too, yep. I would, if something's wrong, that baby's crying, get some advice or something. Talk to someone a little more experienced than you are. (Wiley et al., 2020)

I had a great support system with friends and family, so people helped and cooked meals. (Cox & Roos, 2008)

Papers that mentioned parents who did not have social support indicated that these parents struggled (Ellett et al., 2005, 2009; Kurth et al., 2014; Poskey et al., 2014).

I basically had no help; I was on the verge of quitting everything. (Ellett et al., 2005)

Support from others was useful in multiple ways; it gave parents encouragement and advice, a chance for a break from their baby when people looked after them, and simply, a ‘shoulder to cry on’ (Cox & Roos, 2008; Ellett et al., 2005, 2009; Keefe & Froese‐Fuetz, 1991; Landgren & Hallström, 2011; Landgren et al., 2012; Long & Johnson, 2001; Megel et al., 2011; Murray et al., 2018; Nash et al., 2008; Oaten et al., 2019; Wade et al., 2005; Wiley et al., 2020).

Whilst social support was evidently important for participants of different cultures, some communities use it in different ways. Whilst in the studies from Europe most parents lived alone with their child, the Vietnamese study (Murray et al., 2018) showed how the intergenerational household context meant grandparents were not only a key source of information about unsettled infant behaviour but were very involved in daily infant care.

Maternal and paternal grandmothers have much experience, so they can soothe him better. (Murray et al., 2018)

##### Normalizing crying

Several papers reported that parents discussed what is, and what is not normal in terms of infant crying. Reasons given for normal infant crying were fatigue, hunger, pain and discomfort, prematurity, reflection of environment, the need for social interaction, and understanding that sometimes babies cry for unknown reasons (Cox & Roos, 2008; Ellett et al., 2009; Keefe & Froese‐Fuetz, 1991; Kidd et al., 2019; Kurth et al., 2010; Murray et al., 2018; Thompson et al., 1986).

I kept saying to people that it was because he was premature, this is normal and it will be fine (Cox & Roos, 2008)

In addition, some participants from Vietnam (Murray et al., 2018) had spiritual beliefs about why their babies cried.

It was believed that (…) babies may see or be unsettled by ghosts or ancestors that have passed away. (Murray et al., 2018)

Parents described how they interpreted their baby's cries, and reasons for the crying, and responded accordingly (Cox & Roos, 2008; Ellett et al., 2009; Ellett & Swenson, 2005; Keefe & Froese‐Fuetz, 1991; Kidd et al., 2019; Kurth et al., 2010, 2014; Landgren & Hallström, 2011; Landgren et al., 2012; Levitzky & Cooper, 2000; Long & Johnson, 2001; Murray et al., 2018; Nash et al., 2008; Poskey & Hersch, 2012; Thompson et al., 1986; Wade et al., 2005; Wiley et al., 2020).

In many of the papers, parents' responses were described as being based on characteristics of the crying (Cox & Roos, 2008; Ellett et al., 2009; Keefe & Froese‐Fuetz, 1991; Kidd et al., 2019; Kurth et al., 2010; Landgren et al., 2012; Levitzky & Cooper, 2000; Murray et al., 2018; Nash et al., 2008; Wade et al., 2005; Wiley et al., 2020).

In the beginning it always sounded the same when she was crying. Then I realised she cries differently if she needs to burp or if her nappy is full. I didn't think you could distinguish this, but in fact you can. (Kurth et al., 2014)

Some parents discussed their belief that their infant's crying was not normal, and they believed there was an underlying pathology causing their excessive crying. Parents' search for a diagnosis or cure is discussed later in this paper. Many papers mentioned that parents often believed abnormal crying was caused by something they did wrong, which caused the parents’ emotional distress (Ellett et al., 2005, 2009; Ellett & Swenson, 2005; Keefe & Froese‐Fuetz, 1991; Kurth et al., 2010; Landgren & Hallström, 2011; Murray et al., 2018; Nash et al., 2008; Poskey et al., 2014; Thompson et al., 1986; Wade et al., 2005).

It's like, what's wrong with me or my kid? Why is it this way? Why can't I quiet this baby? (Keefe & Froese‐Fuetz, 1991)

This belief that crying was due to something the parents did arose from parents in almost all papers. Parental beliefs about why they were unable to soothe the baby included ‘bad parenting’, and parents often believed that personal factors, such as maternal stress or anxiety, caused the crying (Ellett et al., 2005, 2009; Ellett & Swenson, 2005; Keefe & Froese‐Fuetz, 1991; Kurth et al., 2010; Landgren & Hallström, 2011; Murray et al., 2018; Nash et al., 2008; Poskey et al., 2014; Thompson et al., 1986; Wade et al., 2005). Some papers also reported that the belief crying represented an underlying pathology was more common in inexperienced parents than those that had experience (Ellett & Swenson, 2005; Kidd et al., 2019; Kurth et al., 2014; Thompson et al., 1986): As one author described, “in contrast to first‐time mothers, experienced mothers understood that all infants had ‘uneasy phases’ and were not always happy. Inexperienced mothers tended to interpret crying as a sign of alarm” (Kurth et al., 2014). Participants in several papers discussed the role of maternal diet and infant feeding. This is explored in theme four of the current paper.

#### 4.2.3 The role of health professionals

##### Health professional support

In many studies, parents described consulting health professionals for their infant's crying, often for support and reassurance that there are no serious underlying issues and that parenting factors are not to blame (Cox & Roos, 2008; Ellett et al., 2005, 2009; Kidd et al., 2019; Kurth et al., 2010; Landgren & Hallström, 2011; Landgren et al., 2012; Levitzky & Cooper, 2000; Long & Johnson, 2001; Megel et al., 2011; Oaten et al., 2019; Thompson et al., 1986; Wade et al., 2005). Parents in a few studies described feeling listened to, felt the health professional understood their situation and had reported having helpful experiences (Cox & Roos, 2008; Ellett et al., 2005; Landgren & Hallström, 2011; Long & Johnson, 2001; Oaten et al., 2019).

He actually listened to me and expressed concern about her colic. He gave us literature on ways to try and calm her down and reasons they believe she is this way. I left the office feeling reassured. She was going to be fine. It was just going to take some time. (Ellett et al., 2005)

Parents' descriptions of positive experiences of health professional support included health visitors (Long & Johnson, 2001; Oaten et al., 2019), midwives (Cox & Roos, 2008), nurses (Landgren & Hallstrom, 2011) and paediatricians (Ellett et al., 2005) and were described as having someone who understands what you are going through, was willing and able to listen and ‘hold your hand’. Parents from the UK (Long & Johnson, 2001; Oaten et al., 2019) particularly valued health visitor support and felt they were able to provide helpful information and reassurance.

What helped was my midwife. Although I'm a logical person, when you're so sleep deprived, you can't even think for yourself; you need somebody literally to hold your hand. (Cox & Roos, 2008)

My health visitor was brilliant. I mean, as far as they can do. They can only offer to listen to you, but that made such a change. She would listen all day if I carried on. (Long & Johnson, 2001)

However, most studies where parents went to health professionals reported that parents felt dismissed, unsupported or did not receive the information they were looking for (Ellett et al., 2005; Keefe & Froese‐Fuetz, 1991; Kurth et al., 2010; Landgren & Hallström, 2011; Megel et al., 2011; Thompson et al., 1986; Wade et al., 2005). As one study author describes “one mother spoke of how, after a few office visits with her colicky infant, her paediatrician started relaying information through the office staff, leaving the mother with the impression that she was an annoyance” (Levitzky & Cooper, 2000)

The nurse said it was normal for babies to cry. She said that colic cannot start as early as the fifth day. But my child did have colic that early, so that was our reality. (Landgren et al., 2012)

##### Seeking a cure

More than half of included papers described parents' search for a treatment or cure for their child's crying (Cox & Roos, 2008; Drummond et al., 1993; Keefe & Froese‐Fuetz, 1991; Kidd et al., 2019; Landgren & Hallström, 2011; Landgren et al., 2012; Long & Johnson, 2001; Megel et al., 2011; Murray et al., 2018; Oaten et al., 2019; Poskey et al., 2014; Poskey & Hersch, 2012; Thompson et al., 1986). Papers that did not discuss seeking a cure were mostly in populations of parents with normal crying infants (Kurth et al., 2010, 2014; Nash et al., 2008; Wade et al., 2005; Wiley et al., 2020). Papers with colicky infants where a cure was not discussed were mostly from one author and specifically focussed on the impact of crying (Ellett et al., 2005, 2009; Ellett & Swenson, 2005; Levitzky & Cooper, 2000).

Parents demonstrated desperation for a treatment or cure and talked about trying various medications, feeding approaches and alternative therapies.

We started our cycle of chiropractors, reflexologists, homeopaths, medication for reflux; we changed his formula a hundred times; we have a hundred bottles, a hundred teats; it was this desperate clinging to something (Cox & Roos, 2008)

You try anything. If anybody can give you a bit of advice that you think you can use, anything at all, you do it. Problem is, of course, none of it works and you're left no better off, maybe a bit poorer, and sometimes it may even seem worse than before. It, like, knocks you back when you build up your hopes and then it doesn't work (Long & Johnson, 2001)

Participants also described their need for more information about infant crying. A few studies specifically described that parents need information about potential reasons for infant crying, and ways of managing it (Drummond et al., 1993; Ellett et al., 2009; Poskey et al., 2014). In one study of parents at risk of abusive head trauma (Wade et al., 2005), a parent disclosed having information about infant crying early on would have enabled them to prepare better and may have affected their decision to have a baby.

I think what would help people, especially who haven't had kids before, is more talk about it in the hospital (…) it's touched on in books (…) they don't really address it (….) I just don't think there's enough awareness… or warning about it. (Ellett et al., 2009)

A parent in one study (Landgren & Hallstrom, 2011) also described searching for information online and feeling like they knew more about colic than the health professionals they were consulting.

I have often felt that I know more than them, at [child health clinic]. They say the same things that they have said for years. I have found new information on the internet, new since my first child had colic six years ago. (Landgren & Hallstrom, 2011)

Seeking a cure or treatment was less likely to emerge from studies where parents did not report a diagnostic label such as colic or excessive crying.

#### 4.2.4 The role of infant feeding and maternal diet

##### Changes to maternal diet

Five papers discussed the belief that maternal diet can cause excessive crying in breastfed babies due to dietary intolerances causing abdominal discomfort and pain (Kidd et al., 2019; Landgren & Hallström, 2011; Levitzky & Cooper, 2000; Megel et al., 2011; Thompson et al., 1986). Babies in all these studies were labelled as having colic or excessive crying. In two papers (Landgren & Hallström, 2011; Thompson et al., 1986), the influence of maternal diet on infant crying emerged only as recommendations from the authors rather than participants. For example, “To gain trust, nurses should give evidence‐based recommendations, like recommending systematic exclusion of cow's milk protein for 5 days as this intervention relieves the symptoms in 5–25% of babies with colic. By guiding parents how to find food for the breastfeeding mother, and formulas free from cow's milk for the bottle‐fed baby, the nurse can make this intervention manageable” (Landgren & Hallstrom, 2011).

In one study (Levitzky & Cooper, 2000), authors described how parents' beliefs and concerns about the impact of maternal diet on infant crying were a result of questioning and advice from health professionals. The authors state “Continuous questions by the pediatrician about the nursing mother's diet often led mothers to believe that their food selection affected the breast milk and therefore caused their infants pain. This compounded mothers' concerns that they were ‘spoiling’ their milk and making their babies sick” (Levitzky & Cooper, 2000).

Data from participants discussing changes to the maternal diet in response to infant crying was only briefly mentioned in one paper (Megel et al., 2011) and extensively discussed in a paper published in 2019 (Kidd et al., 2019), suggesting this concern may be a more recent phenomenon.

The only thing I could have was meat, potatoes, and Italian bread. (Megel et al., 2011)

I have cut out dairy. I have cut out all gassy vegetables and gassy fruits (…) caffeine, and carbonated beverages. Eggs, and, of course, butter. (Kidd et al., 2019)

Parents of formula‐fed infants in two papers discussed changing their baby's formulas as a strategy to reduce colic or persistent crying (Cox & Roos, 2008; Megel et al., 2011).

We changed his formula a hundred times; we have a hundred bottles, a hundred teats; it was this desperate clinging to something (Cox & Roos, 2008)

##### Pressure to stop breastfeeding

Parents of breastfed babies talked about feeling pressured to stop breastfeeding (Keefe & Froese‐Fuetz, 1991; Kidd et al., 2019; Megel et al., 2011; Murray et al., 2018). Often this pressure came from family members suggesting that the baby was crying because breastmilk was insufficient.

Even my husband was like, “Seriously now, when are we going to have that conversation about you not breastfeeding anymore?… I am serious. When are we going to stop hearing a screaming baby? When are we going to get him on formula? (Kidd et al., 2019).

There was also a belief that feeding infants formula milk instead of breastmilk will help them sleep for longer (Kidd et al., 2019; Murray et al., 2018)^.^

He was breastfed for two hours, but he was still crying. I didn't know how to stop his crying, meanwhile two grandmothers were convincing me that feeding him with formula milk can help him fall asleep. I did and still feel regret about that (Murray et al., 2018).

## 5 DISCUSSION

This evidence synthesis reveals the complexity and multifaceted impact of infant crying. The review highlights the wide range of management techniques and coping strategies parents use to deal with infant crying and the importance of social and health professional support. Findings show that parents often have difficulty interpreting why their infant is crying and suspect an abnormal cause, which may lead to overmedicalization. Included studies suggest that a lack of understanding about normal crying often led to intense emotional stress, which had adverse consequences such as social isolation (Cox & Roos, 2008; Ellett et al., 2005, 2009; Keefe & Froese‐Fretz, 1991; Landgren & Hallstrom, 2011; Landgren et al., 2012; Levitzky & Cooper, 2000; Long & Johnson, 2001; Megel et al., 2011; Oaten & Miller, 2019; Poskey, 2012), exhaustion (Cox & Roos, 2008; Ellett et al., 2005, 2009; Kurth et al., 2010; Landgren & Hallstrom, 2011; Landgren et al., 2012; Levitzky & Cooper, 2000; Long & Johnson, 2001; Nash et al., 2008; Oaten & Miller, 2019; Thompson, 2009) and depression (Ellett et al., 2009; Poskey, 2012; Keefe & Froese‐Fretz, 1991; Levitzky & Cooper, 2000). These findings are consistent with a previous literature review of mostly quantitative literature focussing on the consequences of excessive infant crying, which found it to be harmful to relationships and health (Botha et al., 2019). This current review synthesized the qualitative literature and identified parental experiences that go beyond the impact of excessive crying, such as management strategies, the role of health professionals and other support needs and parental help‐seeking behaviours.

Participants in nearly all the papers believed that their child's crying was in some way their fault. They also discussed the importance of social support to help them through that challenging time, yet many parents described feeling judged by others and several paper authors reflected that parents isolate themselves from their social networks in response to unsolicited advice and negative comments. Arming parents with information and coping strategies to respond to perceived judgement may help build and maintain support networks.

Many families described desperately seeking a cure and often consulted health professionals repeatedly for help and support. Findings showed that many parents found their experiences with healthcare professionals to be unhelpful. Parents who reported positive experiences with health professionals valued physical examination of their baby to provide reassurance and rule out underlying causes. Parents also valued feeling understood and listened to by health professionals and being given advice on coping strategies as well as signposting to resources for further support (Cox & Roos, 2008; Ellett et al., 2009; Keefe & Froese‐Fretz, 1991; Landgren & Hallstrom, 2011; Landgren et al., 2012; Long & Johnson, 2001; Megel et al., 2011; Oaten & Miller, 2019).

This review identified beliefs about the impact of maternal diet and infant feeding on excessive crying, considering parental views, and the role of health professionals and other family members. The current findings suggest beliefs that breastmilk is insufficient or harmful to their baby undermines confidence in breastfeeding for parents, families and health professionals. Similar findings were reported in a systematic review of barriers and facilitators to breastfeeding in the first 6 months of life, which found perceptions of insufficient breastmilk to be a significant barrier to exclusive breastfeeding (Balogun et al., 2015). Interestingly, the influence of maternal diet and infant feeding on infant crying was rarely a focus in the qualitative literature and was only discussed by participants in a small minority of papers. Future research is needed to further explore parental beliefs and experiences related to diet and infant crying.

Frustration caused by crying was found to sometimes lead to feelings of anger and sometimes violent intrusive thoughts directed towards the infant (Cox & Roos, 2008; Ellett & Swenson, 2005; Kurth et al., 2010; Landgren et al., 2012; Levitzky & Cooper, 2000; Megel et al., 2011; Nash et al., 2008; Oaten & Miller, 2019; Poskey et al., 2014; Thompson et al., 1986). Current findings further our understanding of how parents experience and manage their frustration, particularly in safety planning, but it does not suggest ways to identify those parents at risk and further research is needed in that area.

Parents' views of crying and ways of managing it were similar across papers despite the different cultural and healthcare contexts of included countries, suggesting a degree of universality across cultures when responding to infant crying. Labels used to describe infant crying ranged across the studies and included ‘colic’, ‘persistent crying’, ‘inconsolable crying’ and ‘excessive crying’. Interestingly, in papers where ‘colic’ was used to describe their participants' infants, this was based on health professional diagnosis by all but one author (Cox & Roos, 2008; Keefe & Froese‐Fuetz, 1991; Landgren & Hallström, 2011; Landgren et al., 2012; Levitzky & Cooper, 2000; Thompson et al., 1986). Studies that described infant crying as ‘persistent’, ‘inconsolable’ or ‘excessive’ were based on parent self‐report (Long & Johnson, 2001; Megel et al., 2011; Oaten et al., 2019; Poskey et al., 2014; Poskey & Hersch, 2012). Most themes arose across all populations, irrespective of the diagnostic label, suggesting the findings are relevant to all parents/carers who consider their infant's crying problematic.

Included studies focused on different samples of participants, such as fathers, grandmothers, and low‐income mothers. These were only a small proportion of the pooled sample with a total of three grandmothers included and seven mothers identified as low‐income. Most included studies did not report the ethnic background of participants and it was not possible to explore how experiences may have differed specifically for fathers, grandparents, low‐income families, or ethnic minority groups. Furthermore, all participants were biologically related to the infants and may not reflect the experiences of alternative family structures. This review focussed on a generalized population of parents so findings may not apply to populations such as parents with mental health conditions.

## 6 CONCLUSION

Infant crying has a major impact on families. Parents use a range of strategies to interpret and deal with the challenges of infant crying, but there is a large unmet need for better resources and support for parents living with excessive infant crying. This review can help health professionals understand the complexity and impact of living with infant crying. Health professionals could support families by ensuring their concerns are listened to and where possible providing reassurance or appropriate diagnoses. Greater awareness of the impact of infant crying such as emotional stress, social isolation, undermined confidence in breastfeeding and feelings of anger and intrusive thoughts could help health professionals provide better and more targeted support and advice when needed. Parents are often desperate for reliable information or a treatment/cure and this needs to be managed with evidence‐based advice and acknowledgement of the impact infant crying can have.

Understanding that parents often feel to blame for their infant's crying may also help health professionals structure supportive discussions. Little is known about the potential effects infant crying may have on parent–child bonding or the impact of dietary modifications or other interventions made in response to infant crying. Further research is needed to explore these potential impacts. Further research is also needed to identify parents in need of support and to find how and when families could be best supported with infant crying.

## AUTHOR CONTRIBUTIONS

M.S., J.M., I.M. and D.G. made substantial contributions to the conception and design of the study. All authors contributed to the acquisition, analysis and interpretation of data. All authors were involved in drafting the manuscript and revising it critically for important intellectual content. All authors also gave final approval of the version to be published and agreed to be accountable for all aspects of the work.

## FUNDING INFORMATION

This research received no specific grant from any funding agency in the public, commercial or not‐for‐profit sectors. The protocol for this systematic review was registered on PROSPERO (CRD42020213056) on 14 October 2020.

## CONFLICT OF INTEREST

No conflict of interest has been declared by the authors.

### PEER REVIEW

The peer review history for this article is available at https://publons.com/publon/10.1111/jan.15492.

## Supporting information


Table S1

Table S2

## Data Availability

Data sharing not applicable to this article as no datasets were generated or analysed during the current study.
