# Supplementary material for: Parental perceptions and experiences of infant crying: A systematic review and synthesis of qualitative research
Source: J Adv Nurs. 2022 Nov 14;79(2):403–17. doi: 10.1111/jan.15492 (PMC10100257; doi:10.1111/jan.15492)
Supplement: Supplementary file 1 — Table S1 [file JAN-79-403-s001.docx]

**SUPPLEMENTARY MATERIALS**

**Table S1: Comprehensive search strategies for each database**

1. **EMBASE**

| **Search ID #** | **Search terms** | |
| --- | --- | --- |
| 1 | Infant/ or infant*.ti,ab,kf. | |
| 2 | Baby*.ti,ab,kf. | |
| 3 | Babies*.ti,ab,kf. | |
| 4 | Newborn*.ti,ab,kf. | |
| 5 | Neonate*.ti,ab,kf. | |
| 6 | 1 or 2 or 3 or 4 or 5 | |
| 7 | Parent-child relations/ | |
| 8 | Parents/ or parent*.ti,ab,kf. | |
| 9 | Mothers/ or mother*.ti,ab,kf. | |
| 10 | Fathers/ or father*.ti,ab,kf. | |
| 11 | Caregiver*.ti,ab,kf. | |
| 12 | Maternal*.ti,ab,kf. | |
| 13 | Paternal*.ti,ab,kf. | |
| 14 | Parental*.ti,ab,kf. | |
| 15 | 7 or 8 or 9 or 10 or 11 or 12 or 13 or 14 | |
| 16 | Crying/ or cry*.ti,ab,kf. | |
| 17 | Colic/ or colic*.ti,ab,kf. | |
| 18 | Infant behavior/ or infant behavio*.ti,ab,kf. | |
| 19 | Fuss*.ti,ab,kf. | |
| 20 | Irritab*.ti,ab,kf. | |
| 21 | Unsettl*.ti,ab,kf. | |
| 22 | 16 or 17 or 18 or 19 or 20 or 21 | |
| 23 | Qualitative research/ or qualitative.mp. | |
| 24 | Interview/ | |
| 25 | Focus groups/ or focus group*.mp. | |
| 26 | Observation/ or observation*.mp. | |
| 27 | Ethnograph*.mp. | |
| 28 | Grounded theory.mp. | |
| 29 | Phenomenol*.mp. | |
| 30 | Content analysis.mp. | |
| 31 | Thematic analysis.mp. | |
| 32 | Attitude*.mp. | |
| 33 | Discourse*.mp. | |
| 34 | Experience*.mp. | |
| 35 | Narrative*.mp. | |
| 36 | 23 or 24 or 25 or 26 or 27 or 28 or 29 or 30 or 31 or 32 or 33 or 34 or 35 | |
| 37 | 6 and 15 and 22 and 36 | |
| Embase (Ovid) 1974 to 2022 January | |  |
| Search date 28 January 2022 | |  |

1. **MEDLINE**

| **Search ID #** | **Search terms** |
| --- | --- |
| 1 | Infant/ or infant*.ti,ab,kf. |
| 2 | Baby*.ti,ab,kf. |
| 3 | Babies*.ti,ab,kf. |
| 4 | Newborn*.ti,ab,kf. |
| 5 | Neonate*.ti,ab,kf. |
| 6 | 1 or 2 or 3 or 4 or 5 |
| 7 | Parent-child relations/ |
| 8 | Parents/ or parent*.ti,ab,kf. |
| 9 | Mothers/ or mother*.ti,ab,kf. |
| 10 | Fathers/ or father*.ti,ab,kf. |
| 11 | Caregiver*.ti,ab,kf. |
| 12 | Maternal*.ti,ab,kf. |
| 13 | Paternal*.ti,ab,kf. |
| 14 | Parental*.ti,ab,kf. |
| 15 | 7 or 8 or 9 or 10 or 11 or 12 or 13 or 14 |
| 16 | Crying/ or cry*.ti,ab,kf. |
| 17 | Colic/ or colic*.ti,ab,kf. |
| 18 | Infant behavior/ or infant behavio*.ti,ab,kf. |
| 19 | Fuss*.ti,ab,kf. |
| 20 | Irritab*.ti,ab,kf. |
| 21 | Unsettl*.ti,ab,kf. |
| 22 | 16 or 17 or 18 or 19 or 20 or 21 |
| 23 | Qualitative research/ or qualitative.mp. |
| 24 | Interview/ |
| 25 | Focus groups/ or focus group*.mp. |
| 26 | Observation/ or observation*.mp. |
| 27 | Ethnograph*.mp. |
| 28 | Grounded theory.mp. |
| 29 | Phenomenol*.mp. |
| 30 | Content analysis.mp. |
| 31 | Thematic analysis.mp. |
| 32 | Attitude*.mp. |
| 33 | Discourse*.mp. |
| 34 | Experience*.mp. |
| 35 | Narrative*.mp. |
| 36 | 23 or 24 or 25 or 26 or 27 or 28 or 29 or 30 or 31 or 32 or 33 or 34 or 35 |
| 37 | 6 and 15 and 22 and 36 |

Embase (Ovid) 1974 to 2022 January

Search date 28 January 2022

1. **PsycINFO**

| **Search ID #** | **Search terms** |
| --- | --- |
| S1 | (TI(infant* or baby* or babies* or newborn* or neonate*)) OR (AB(infant* or baby* or babies* or newborn* or neonate*)) OR (KW(infant* or baby* or babies* or newborn* or neonate*)) |
| S2 | DE "Parent Child Relations" OR DE "Father Child Relations" OR DE "Mother Child Relations" OR DE "Parental Attitudes" |
| S3 | (TI(parent* or mother* or father* or caregiver* or maternal* or paternal* or parental*)) OR (AB(parent* or mother* or father* or caregiver* or maternal* or paternal* or parental*)) OR (KW(parent* or mother* or father* or caregiver* or maternal* or paternal* or parental*)) |
| S4 | S2 OR S3 |
| S5 | DE "Crying" |
| S6 | (TI(cry* or colic* or infant behavio* or fuss* or irritab* or unsettl*)) OR (AB(cry* or colic* or infant behavio* or fuss* or irritab* or unsettl*)) OR (KW(cry* or colic* or infant behavio* or fuss* or irritab* or unsettl*)) |
| S7 | S5 OR S6 |
| S8 | DE "Qualitative Methods" OR DE "Focus Group" OR DE "Grounded Theory" OR DE "Interpretative Phenomenological Analysis" OR DE "Narrative Analysis" OR DE "Semi-Structured Interview" OR DE "Thematic Analysis" |
| S9 | DE "Interviews" |
| S10 | DE "Observation Methods" |
| S11 | DE "Phenomenology" |
| S12 | (TI(ethnograph* or grounded theory or phenomenol* or content analysis or thematic analysis or discourse* or narrative* or attitude*)) OR (AB(ethnograph* or grounded theory or phenomenol* or content analysis or thematic analysis or discourse* or narrative* or attitude*)) OR (KW(ethnograph* or grounded theory or phenomenol* or content analysis or thematic analysis or discourse* or narrative* or attitude*)) |
| S13 | S8 OR S9 OR S10 OR S11 OR S12 |
| S14 | S1 AND S4 AND S7 AND S13 |
|  |  |
|  | PsycINFO (EbscoHOST) |
|  | Search date 28 January 2022 |

1. **CINAHL**

| **Search ID #** | **Search terms** |  |  |
| --- | --- | --- | --- |
| S1 | (MH "Infant") |  |  |
| S2 | (TI(infant* or baby* or babies* or newborn* or neonate*)) OR (AB(infant* or baby* or babies* or newborn* or neonate*)) |  |  |
| S3 | S1 OR S2 |  |  |
| S4 | (MH "Parent-Infant Relations") |  |  |
| S5 | (MH "Parents") |  |  |
| S6 | (MH "Mothers") |  |  |
| S7 | (MH "Fathers") |  |  |
| S8 | (TI(parent* or mother* or father* or caregiver* or maternal* or paternal* or parental*)) OR (AB(parent* or mother* or father* or caregiver* or maternal* or paternal* or parental*)) |  |  |
| S9 | S4 OR S5 OR S6 OR S7 OR S8 |  |  |
| S10 | (MH "Crying") |  |  |
| S11 | (MH "Infant Colic") |  |  |
| S12 | (MH "Infant Behavior") |  |  |
| S13 | (TI(cry* or colic* or infant behavio* or fuss* or irritab* or unsettl*)) OR (AB(cry* or colic* or infant behavio* or fuss* or irritab* or unsettl*)) |  |  |
| S14 | S10 OR S11 OR S12 OR S13 |  |  |
| S15 | (MH "Qualitative Studies") |  |  |
| S16 | (MH "Interviews") |  |  |
| S17 | (MH "Focus groups") |  |  |
| S18 | (MH "Observational Methods") |  |  |
| S19 | (MH "Ethnographic Research") |  |  |
| S20 | (MH "Grounded Theory") |  |  |
| S21 | (MH "Phenomenological Research") |  |  |
| S22 | (MH "Content Analysis") |  |  |
| S23 | (MH "Thematic Analysis") |  |  |
| S24 | (MH "Discourse Analysis") |  |  |
| S25 | (MH "Narratives") |  |  |
| S26 | qualitative |  |  |
| S27 | (TI(ethnograph* or grounded theory or phenomenol* or content analysis or thematic analysis or discourse* or narrative* or attitude*)) OR (AB(ethnograph* or grounded theory or phenomenol* or content analysis or thematic analysis or discourse* or narrative* or attitude*)) |  |  |
| S28 | S15 OR S16 OR S17 OR S18 OR S19 OR S20 OR S21 OR S22 OR S23 OR S24 OR S25 OR S26 OR S27 |  |  |
| S29 | S3 AND S9 AND S14 AND S28 |  |  |
|  |  |  |  |
|  |  |  |  |
|  | CINAHL (EbscoHOST) |  |  |
|  | Search date 28 January 2022 |  |  |
