# Supplementary material for: Parental perceptions and experiences of infant crying: A systematic review and synthesis of qualitative research
Source: J Adv Nurs. 2022 Nov 14;79(2):403–17. doi: 10.1111/jan.15492 (PMC10100257; doi:10.1111/jan.15492)
Supplement: Supplementary file 2 — Table S2 [file JAN-79-403-s002.docx]

Supplementary materials

**Table S2: Quality appraisal of included papers: CASP results**

| **Study reference** | Is this study qualitative research? | Are the research questions clearly stated? | Is the qualitative approach clearly justified? | Is the study context clearly described? | Is the role of the researcher clearly described? | Is there a clear connection to an existing body of knowledge/ wider research? | Is the sampling method clearly described? | Is the sampling strategy appropriate for the research question? | Is the method of data collection clearly described? | Is the data collection method appropriate to the research question? | Is the method of data analysis clearly described? | Is the data analysis method appropriate to the research question? | Are the claims made supported by sufficient evidence? | Comment |
| --- | --- | --- | --- | --- | --- | --- | --- | --- | --- | --- | --- | --- | --- | --- |
| Thompson et al (1986) | Yes | Unclear | Yes | Yes | No | Yes | No | No | Unclear | No | Yes | No | Unsure | Low quality paper |
| Wiley et al (2020) | Yes | Yes | Yes | Yes | Yes | Yes | Yes | Yes | Yes | Yes | Yes | Yes | Yes | Very high quality paper |
| Wade et al (2005) | Yes | Yes | Yes | Yes | Yes | Yes | Yes | Yes | Yes | Yes | Yes | Yes | Yes | Very high quality paper |
| Landgren et al (2012) | Yes | Yes | Yes | Yes | Yes | Yes | Yes | Yes | Yes | Yes | Yes | Yes | Yes | Very high quality paper |
| Cox et al (2008) | Yes | Yes | Yes | Yes | Yes | Yes | Yes | Yes | Yes | Yes | Yes | Yes | Yes | Very high quality paper |
| Drummond et al (1993) | Yes | Yes | Yes | Yes | Yes | Yes | Yes | Yes | Yes | Yes | Yes | Yes | Yes | High quality paper |
| Kurth et al (2010) | Yes | Yes | Yes | Yes | Yes | Yes | Yes | Yes | Yes | Yes | Yes | Yes | Yes | Very high quality paper |
| Landgren et al (2011) | Yes | Yes | Yes | Yes | Yes | Yes | Yes | Yes | Yes | Yes | Yes | Yes | Yes | Very high quality paper |
| Megel et al (2011) | Yes | Yes | Yes | Yes | Yes | Yes | Yes | Yes | Yes | Yes | Yes | Yes | Yes | Very high quality paper |
| Murray et al (2018) | Yes | Yes | Yes | Yes | Yes | Yes | Yes | Yes | Yes | Yes | Yes | Yes | Yes | Very high quality paper |
| Poskey et al (2012) | Yes | Yes | Yes | Yes | Yes | Yes | Yes | Yes | Yes | Yes | Yes | Yes | Yes | Very high quality paper |
| Poskey et al (2014) | Yes | Yes | Yes | Yes | Yes | Yes | Yes | Yes | Yes | Yes | Yes | Yes | Yes | Very high quality paper |
| Ellett et al (2009) | Yes | Yes | Yes | Yes | Yes | Yes | Yes | Yes | Yes | Yes | Yes | Yes | Yes | Very high quality paper |
| Keefe et al (1991) | Yes | Yes | Yes | Yes | Yes | Yes | Yes | Yes | Yes | Yes | Yes | Yes | Yes | Very high quality paper |
| Kidd et al (2019) | Yes | Yes | Yes | Yes | Yes | Yes | Yes | Yes | Yes | Yes | Yes | Yes | Yes | Very high quality paper |
| Ellett et al (2005) | Yes | Yes | Yes | Yes | Yes | Yes | Yes | Yes | Yes | Yes | Yes | Yes | Yes | Very high quality paper |
| Nash et al (2008) ^22^ | Yes | Yes | Yes | Yes | Partly- doesn’t state area | Yes | Yes | Yes | Yes | Yes | Yes | Yes | Yes | High quality paper |
| Kurth et al (2014) | Yes | Yes | Yes | Yes | Yes | Yes | Yes | Yes | Yes | Yes | Yes | Yes | Yes | Very high quality paper |
| Long et al (2001) | Yes | Yes | Yes | Yes | Yes | Yes | Yes | Yes | Yes | Yes | Yes | Yes | Yes | Very high quality paper |
| Ellett et al (2005) | Yes | Yes | Yes | Yes | Yes | Yes | Yes | Yes | Yes | Yes | Yes | Yes | Yes | Very high quality paper |
| Oaten et al (2019) | Yes | Yes | Yes | Yes | Yes | Yes | Yes | Yes | Yes | Yes | Yes | Yes | Yes | Very high quality paper |
| Levitzky et al (2000) | Yes | Yes | Yes | Yes | Yes | Yes | Yes | Yes | Yes | Yes | Not clearly described | Unsure | Yes | High quality paper |
